# Neural responses to social evaluative threat in the absence of negative investigator feedback and provoked performance failures

**DOI:** 10.1002/hbm.24932

**Published:** 2020-01-20

**Authors:** Phöbe Fehlner, Edda Bilek, Anais Harneit, Andreas Böhringer, Carolin Moessnang, Andreas Meyer‐Lindenberg, Heike Tost

**Affiliations:** ^1^ Department of Psychiatry and Psychotherapy Central Institute of Mental Health, Medical Faculty Mannheim, University of Heidelberg Mannheim Germany

**Keywords:** functional neuroimaging, mental health, risk factors, social perception, stress

## Abstract

Functional neuroimaging of social stress induction has considerably furthered our understanding of the neural risk architecture of stress‐related mental disorders. However, broad application of existing neuroimaging stress paradigms is challenging, among others due to the relatively high intensity of the employed stressors, which limits applications in patients and longitudinal study designs. Here, we introduce a less intense neuroimaging stress paradigm in which subjects anticipate, prepare, and give speeches under simulated social evaluation without harsh investigator feedback or provoked performance failures (IMaging Paradigm for Evaluative Social Stress, IMPRESS). We show that IMPRESS significantly increases perceived arousal as well as adrenergic (heart rate, pupil diameter, and blood pressure) and hormonal (cortisol) responses. Amygdala and perigenual anterior cingulate cortex (pACC), two key regions of the emotion and stress regulatory circuitry, are significantly engaged by IMPRESS. We further report associations of amygdala and pACC responses with measures of adrenergic arousal (heart rate, pupil diameter) and social environmental risk factors (adverse childhood experiences, urban living). Our data indicate that IMPRESS induces benchmark psychological and endocrinological responses to social evaluative stress, taps into core neural circuits related to stress processing and mental health risk, and is promising for application in mental illness and in longitudinal study designs.

## 1 INTRODUCTION

The sympathetic nervous system and the hypothalamic–pituitary–adrenal (HPA) axis elicit evolutionarily conserved responses to stress, including increases in heart rate, pupil diameter, blood pressure, and cortisol levels, aiming at mobilizing resources to overcome the perceived threat (Ulrich‐Lai & Herman, 2009). Current evidence suggests that prolonged exposure to social stress and related alterations in neural stress regulatory circuits (McEwen et al., 2015) are at the core of the effects of well‐established social risk factors for stress‐related psychiatric disorders, including adverse childhood experiences (ACE; Teicher, Samson, Anderson, & Ohashi, 2016), urban living (Mortensen et al., 1999), or ethnic minority status (van der Ven & Selten, 2018). Specifically, chronic social stress exposure has been linked (a) to stress‐related psychiatric disorders including for example, depression, anxiety disorders, eating disorders, and psychosis (Hailes, Yu, Danese, & Fazel, 2019) and (b) to structural and functional alterations in brain regions involved in threat appraisal and emotion regulation, in particular in the amygdala and perigenual anterior cingulate cortex (pACC; Dannlowski et al., 2012; Holz, Tost, & Meyer‐Lindenberg, 2019; Lederbogen et al., 2011; Meyer‐Lindenberg & Tost, 2012; Teicher et al., 2016; Tost, Champagne, & Meyer‐Lindenberg, 2015). These stress‐related brain alterations, which can be observed on the molecular (epigenetic), cellular (e.g., glucocorticoid receptor gene expression), and systemic level (structure, function, and interaction of brain regions), are believed to increase the risk for stress‐related psychiatric illness by limiting the ability to cope effectively with acute stress experiences (McEwen, 2004; McEwen et al., 2015; Meyer‐Lindenberg & Tost, 2012; Teicher et al., 2016; Tost et al., 2015; Ulrich‐Lai & Herman, 2009; Zorn et al., 2017). Although the exact underlying mechanisms are still subject to investigation, the effects of diverse risk factors for stress‐related mental disorders seem to converge on structural and functional alterations in the amygdala and pACC in numerous studies (Holz et al., 2019; Meyer‐Lindenberg & Tost, 2012; Tost et al., 2015). This is in line with current models proposing deficient top‐down regulation of the amygdala by pACC in subjects with chronic stress exposure, which may lead to overactivation of the amygdala and consequently increased stress‐related responses, as well as compensatory pACC overactivation and eventually blunted (worn out) cortisol responses (McEwen, 2004; Pezawas et al., 2005; Ulrich‐Lai & Herman, 2009; Zorn et al., 2017).

Functional neuroimaging of social stress induction has become an important topic in psychiatry research since the approach allows for the identification and mechanistic study of aberrant neural responses to social stress in healthy at‐risk populations and psychiatric patients (Meyer‐Lindenberg & Tost, 2012; Tost et al., 2015). Earlier studies have applied a variety of paradigms to induce social stress, prominent examples being fMRI adaptions of the well‐established Trier social stress test (TSST; Kirschbaum, Pirke, & Hellhammer, 1993). Adaptations of the TSST for fMRI typically emphasize perceptions of uncontrollability and social evaluative threat (SET; Dickerson & Kemeny, 2004) by combining increased cognitive task demands, time pressure, and social evaluation with negative performance feedback by the investigators, often with additional provocation of performance failures. For example, several studies have applied arithmetic problems with individually adjusted difficulty at performance limit with an intense negative performance feedback to convince the participants that they perform very poorly (Akdeniz et al., 2014; Dedovic et al., 2005; Dedovic, D'Aguiar, & Pruessner, 2009; Lederbogen et al., 2011; Streit et al., 2014). While these paradigms achieve their goal of strongly activating social stress‐related circuits, the fMRI experiments also bear several inherent limitations (Dedovic et al., 2009). First, stress induction is often confounded with cognitive load (i.e., the stress induction and control conditions differ in more than just social evaluation, e.g., by time pressure or task difficulty). Second, the more immersive the experimental set‐ups, the more prone they typically are to between‐subject variations in stressor manipulation, in particular when live investigator panels are used. Third, the provided intense negative feedback can result in strong physiological and emotional responses of the participants, which limit application in psychiatric patient populations. Finally, the deception component inherent to the provoked performance failures and negative performance feedback requires a thorough debriefing of participants at the end of the task, which makes repeated administration of these paradigms in longitudinal study designs impractical.

These challenges can be addressed by avoiding any feedback and performance control aspects in the task and instead focus on stress experiences induced by the anticipation of (Tillfors, Furmark, Marteinsdottir, & Fredrikson, 2002), preparation for (Wager, van Ast, et al., 2009; Wager, Waugh, et al., 2009), or processing of (Tillfors et al., 2001) social evaluation during public speaking. Neuroimaging studies using this approach often consisted of one or two experimental and control task phases of 2–3 min duration each (Tillfors et al., 2001, 2002; Wager, van Ast, et al., 2009; Wager, Waugh, et al., 2009). For example, Tillfors et al. (2001, 2002) compared speaking on autobiographical experiences alone versus in front of an audience. Wager, van Ast, et al. (2009) and Wager, Waugh, et al. (2009) compared phases of the mental preparation of speeches on participant characteristics or economic topics with phases of resting‐state. These paradigms are considered to induce a certain level of SET to the participant by the mere possibility to be judged unfavorably by others (Dickerson & Kemeny, 2004; Wager, van Ast, et al., 2009; Wager, Waugh, et al., 2009). Aside from fMRI adaptions of the TSST, several other approaches for social stress induction in the MRI environment have been used (Noack, Nolte, Nieratschker, Habel, & Derntl, 2019) such as the socially evaluated cold‐pressor test (SECPT). The SECPT involves social evaluation of how well participants tolerate the exposure of their hand to a painfully cold water stimulus of 0–4°C (Schwabe, Haddad, & Schachinger, 2008) and is often combined with arithmetic tasks (Luo et al., 2018; Smeets et al., 2012; Vogel et al., 2015; Zhang et al., 2019). In contrast to the paradigms described so far, the SECPT is not designed for the online measurement of brain function during acute stress processing, since fMRI data is collected after, and not during, stress induction (Luo et al., 2018; Vogel et al., 2015; Zhang et al., 2019). Also, the social evaluation of pain tolerance in the SECPT may be ecologically less valid than giving speeches in front of an audience.

In this study, we aimed to adapt the TSST scenario to the demands of neuroimaging research, thereby addressing several challenges related to earlier task designs. In the IMaging PaRadigm for Evaluative Social Stress (IMPRESS) task, participants gave free speeches similar to that in job interviews in front of evaluators presented in a video clip. The task conditions differed only in the presence of SET and were standardized across subjects using prerecorded videos of the evaluators. We further refrained from implementing unavoidable performance failures or harsh investigator feedbacks to make the task ecologically more valid and better applicable in patients. Finally, IMPRESS is structured in distinct task phases to investigate and separate the anticipation, preparation, and processing of SET in a single paradigm. We aimed to validate our novel fMRI task by means of physiological and subjective measures of arousal that we assessed online, that is, during neuroimaging (heart rate, pupil diameter, and subjective arousal) and offline (heart rate, blood pressure, and cortisol). We expected to detect higher online arousal levels during the SET condition compared to the control condition and higher offline arousal levels after IMPRESS task runs compared to measurements before the task, respectively. On the level of brain function, we expected to see significant activation increases in the SET condition compared to the control condition in brain regions previously associated with neural social stress processing, in particular in the amygdala and pACC (Akdeniz et al., 2014; Dedovic et al., 2005; Streit et al., 2014). We further aimed to probe the value of our paradigm by relating variation in established measures of arousal (i.e., heart rate and pupil diameter) and social environmental risk for psychiatric disorders (i.e., ACE and urban living) with variation in SET‐associated brain activity. More precisely, we explored whether we could replicate established findings (Gianaros et al., 2005, 2008, 2017; Gianaros, Jennings, Sheu, Derbyshire, & Matthews, 2007; Heany et al., 2018; Lederbogen et al., 2011; Wager, Waugh, et al., 2009) using our novel paradigm. As our previous work linked social environmental risk exposure to amygdala and pACC, we confined our analyses to these two brain regions. Based on our previous findings, as well as on studies that investigated the effect of ACE during emotional face processing, we expected a positive correlation of ACE severity with amygdala (Dannlowski et al., 2012, 2013; Heany et al., 2018) and pACC (Fonzo et al., 2013, 2016; Hart et al., 2018; Heany et al., 2018) activity during social stress processing. Regarding urban living, we aimed to replicate our previous findings, that is, positive associations of (a) current urban living with increased amygdala activity and (b) urban upbringing with increased pACC activity during social stress processing (Lederbogen et al., 2011).

## 2 MATERIALS AND METHODS

### 2.1 Participants

The study conforms to the standards of the Declaration of Helsinki. All participants gave written informed consent prior to participation to a study protocol approved by the ethics committee of the Medical Faculty Mannheim of Heidelberg University. In total, we collected data from 53 participants. To ensure high data quality, a total of 12 subjects were excluded from all analyses: Four subjects were excluded because of insufficient fMRI data quality, that is, spikes or movement artifacts during scanning [total translation > 5.5 mm or > 50 % of volumes exceeding 0.5 mm frame‐by frame head movements (Power, Barnes, Snyder, Schlaggar, & Petersen, 2012) or continuous rhythmic movement artifacts during the performance phase]. Eight subjects were excluded because they reported they had no motivation to give good talks (*N* = 1), did not believe in the simulated live investigator panel (*N* = 4), or did not feel intimidated at all by the panel (*N* = 3). The final sample consisted of 41 healthy volunteers (females: *N* = 24; 59 %) with fluent German language knowledge and a mean (M) age ± standard deviation (*SD*) of 25.2 ± 6.0 years. Participants were generally well‐educated (years of school education: M = 12.7, *SD* = 0.9), right‐handed (*N* = 40), nonsmoking (*N* = 40), and of normal weight (body mass index [kg/m^2^]: M = 23.37, *SD* = 3.73). Disregarding hormonal contraceptives (*N* = 16 females; 39 %), three participants reported medication intake on the day of measurement (nonsteroidal anti‐inflammatory drug, H1‐antihistamine, and 5α‐reductase inhibitor, respectively). Most participants (*N* = 32, 78 %) had previously participated in fMRI studies and were thus familiar with the MR environment. We excluded volunteers in the case of contraindications for MRI, neurological or significant other medical illnesses, and current or personal history of drug abuse or DSM‐IV axis I disorders (SCID‐I).

### 2.2 General protocol

**Figure 1:**
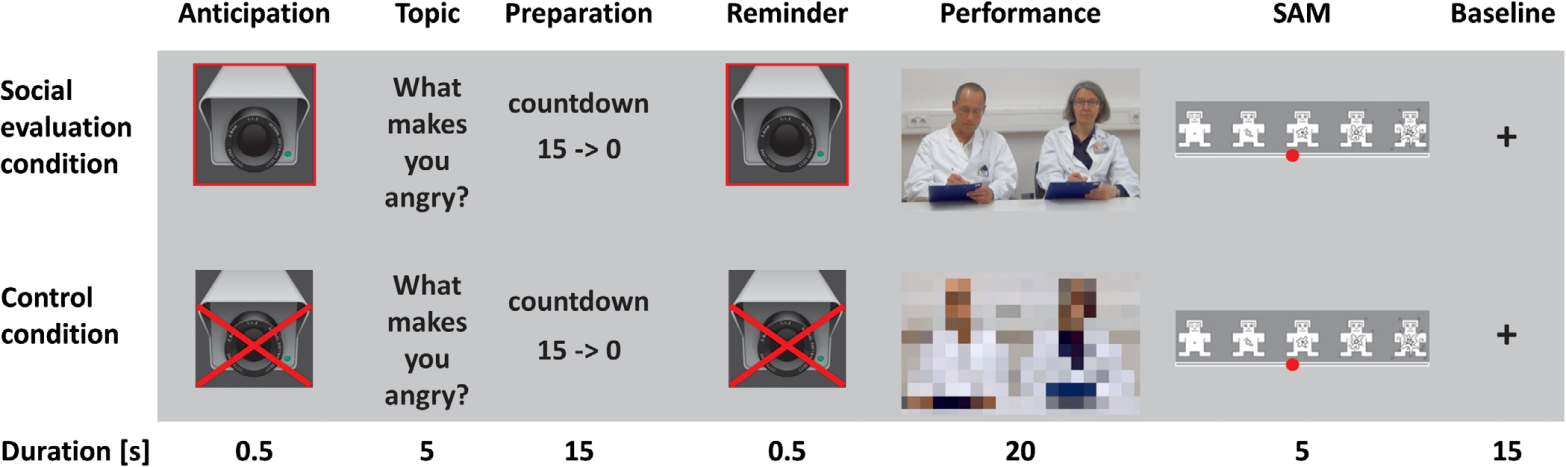
Example trial of the evaluative social stress paradigm. Task phases are depicted in columns in chronological sequence from left to right. Conditions are depicted in rows

Subjects were instructed not to (a) ingest food or caffeine 2 hr prior to study participation, (b) exercise on the study day or the evening before, and (c) sleep during the study day. Data collection took place in the evenings (5–9 p.m.). Prior to fMRI, participants rested in a supine position for 15 min in a quiet room to ensure comparable baseline conditions across subjects. Arousal indicators were collected offline (heart rate, blood pressure, and saliva cortisol; T1 after rest, T2 before fMRI, T3 in between the two fMRI runs, T4 after fMRI, and T5 ~20 min after task completion; see Figure 1 for exact timing) and online during the two fMRI runs [heart rate and pupil diameter continuously and self‐assessment manikin (SAM, Bradley & Lang, 1994) arousal at the end of each trial)].

### 2.3 Evaluative social stress paradigm

During fMRI, participants anticipated, prepared, and delivered free speeches to selected topics. The task conditions differed only in the presence or absence of SET during speech production, that is, in the type of video clips displayed during the speeches. All other task elements were identical for both conditions.

#### 2.3.1 Rater panel simulation and evaluation task condition

Participants were instructed that during the task, a panel of speech rating experts in a nearby office would see and hear them via video‐live‐stream and would thoroughly observe and evaluate the subjects’ speech quality (content, structure, clarity, fluency, sound volume, and facial expressions) during experimental trials. For standardization purposes, video sequences of three distinct panels were prerecorded to ensure that participants were exposed to comparable stimuli. Each panel consisted of one male and one female investigator to avoid confounding effects of panel sex composition (Duchesne, Tessera, Dedovic, Engert, & Pruessner, 2012). Panel members were middle‐aged, wore lab coats, and acted as if they were thoroughly evaluating the participant's performance (by apparently observing the subject, making regular notes on a clipboard, and showing serious and concentrated facial expressions; see Supporting Information for details on video recording and processing). To enhance the credibility of the apparent live evaluation, we used prerecorded video sequences of the panels to simulate live communication with the attending staff immediately before the fMRI task started.

#### 2.3.2 Control condition

In control trials, participants were shown pixelated video footage of the panel during speech production and were told that the panel would not be able to hear or see them, so that the speech would be given in an unobserved situation, similar to a practice trial. Participants were instructed to nonetheless keep the performance level high in the control trials. They were further instructed that the presence or absence of speech output would be automatically tracked by a computer and that in cases where the speech production would go below 20 s in a control trial the data acquisition would be automatically aborted.

#### 2.3.3 Task phases and structure of trials

In total, the paradigm consisted of seven different task phases in each trial (Figure 1). Each trial started with an anticipation phase (1), which informed the participant on the task condition of the upcoming trial, that is, whether the speech in the performance phase would have to be given under social evaluation or not. The anticipation phase was signaled by the 0.5 s display of the picture of a camera (fully visible in the experimental condition, crossed out in the control condition). In the following speech topic presentation phase (2) the speech topic was presented for 5 s (e.g., “What are the attributes of a good leader?”, see Supporting Information for details on the speech topic selection process). In the subsequent speech preparation phase (3), the subjects had 15 s to develop convincing arguments and structure the speech in a logical way, while being exposed to a countdown from 15 to 0 in steps of seconds to induce time pressure. Then, the anticipation phase was repeated as a reminder of the task condition (4). During the performance phase (5), the subject gave the prepared speech for 20 s, while being exposed to a prerecorded video clip of the evaluation panel (pixelated video clip in control trials). Afterward, the subject rated how arousing the trial was on an 11‐point SAM arousal scale (6). Each trial ended with a fixation cross shown for 15 s to temporally separate consecutive trials (7). The paradigm consisted of two runs of 14 trials (~76 s) each, which summed up to a total task duration of approximately 36 min. Notably, within trials, task phases 1 to 6 were temporally separated to reduce the probability of collinearity between the different trial events using five arrays of 14 jitters with identical M and *SD* of 2,977 ± 589 ms.

#### 2.3.4 Randomization of rater panels, speech topics, and order of task conditions

For future potential longitudinal use of the task, each subject was randomly assigned to one out of two panels of raters, one out of two pseudorandomized orders of conditions (the same condition was presented in a maximum of three consecutive trials, both runs contained seven control and seven experimental trials), and one out of two sets of speech topics. Within the set of speech topics, the order of items was randomized for each subject, that is, the same topic was presented within an experimental trial for some subjects and within a control trial for other subjects to ensure that the topic‐related arousal levels did not differ between conditions.

The paradigm was implemented using Presentation® software version 17.2 (https://www.neurobs.com/). During instructions, the sequence of task phases was explained and shown to the subject using the same software and timing as in the actual task. An active noise cancelation system (OptoAcoustics Ltd., Tel‐Aviv, Israel) was used to reduce distraction of subjects by scanner noise.

### 2.4 Acquisition and analysis of arousal data

See Supporting Information for details.

### 2.5 MRI data acquisition and preprocessing

See Supporting Information for details.

### 2.6 FMRI data analysis

The activation analysis of the fMRI data consisted of a two‐level procedure. At the first level, a general linear model (GLM) was defined for each subject. GLMs included the regressors for the different task phases and conditions (stick function: Anticipation and reminder phase, box‐car functions: Performance and preparation phase, respectively, all convolved with the standard SPM canonical hemodynamic response function) and the six head motion parameters from the realignment step as covariates of noninterest. The topic presentation phase was not in the focus of this study and was thus not modeled. During model estimation, the data were high‐pass filtered (cut‐off: 256 s) and individual maps of the contrasts SET > control for the anticipation, preparation, and performance phases were computed.

At the second level, we entered the contrast images of the participants into random‐effects group analyses. We used one‐sample *t* tests to examine the neural effects of SET. Moreover, we calculated linear regression models in which physiological arousal variables [heart rate and pupil diameter: Difference between task conditions, cortisol: Area under the curve with respect to increase (AUCi; Pruessner, Kirschbaum, Meinlschmid, & Hellhammer, 2003)] and questionnaire measures of psychiatric risk exposure were defined as independent variables. Risk variables included total scores of the German version of the childhood trauma questionnaire (CTQ; Wingenfeld et al., 2010), urban upbringing, and current urbanicity (Lederbogen et al., 2011), respectively (see Supporting Information for details). In all second‐level models, sex and age were included as covariates of noninterest. Urban upbringing was entered as an additional covariate of noninterest in the regression model for current urbanicity and vice versa. In line with our a priori hypotheses, we used a combined bilateral mask consisting of the amygdala (Automated Anatomical Labeling atlas; Tzourio‐Mazoyer et al., 2002) and the pACC (empirically defined based on prior work, see Supporting Information for details) for region of interest (ROI) correction. The significance threshold was set to *p* < .05, family‐wise error (FWE) corrected for multiple comparisons within our a priori defined ROI.

## 3 RESULTS

### 3.1 Task‐associated arousal

**Figure 2:**
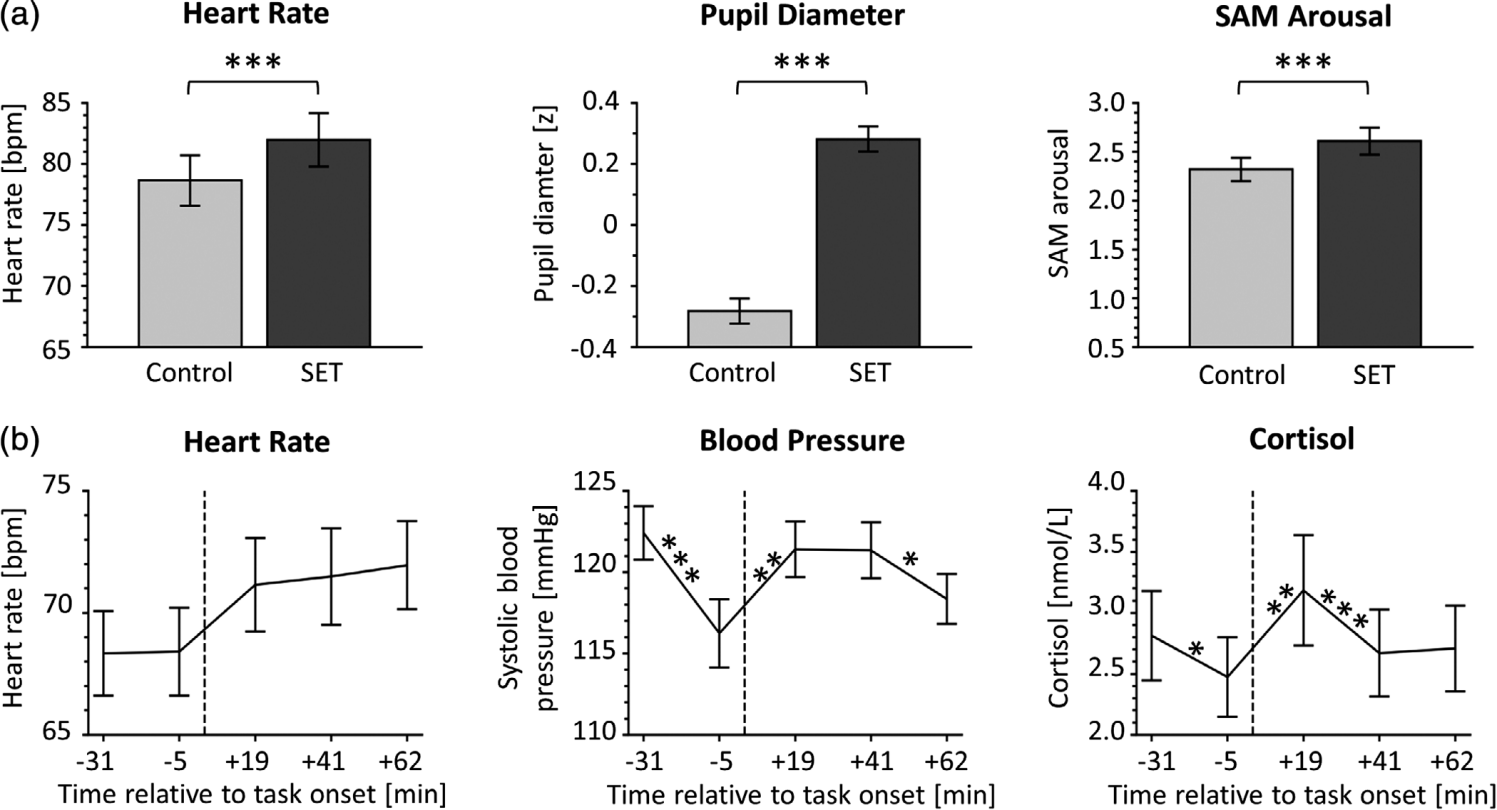
Effects of SET on task‐associated arousal measures. (a) arousal data (mean, M, ± standard error, SE) for the two fMRI task conditions. Heart rate (data missing for five subjects) and pupil diameter were measured continuously and SAM arousal at the end of each fMRI trial. (b) M ± SE for heart rate, blood pressure, and saliva cortisol (data missing for two subjects), measured at five time points and reported relative to the onset of fMRI Run 1 (dashed line). bpm, beats per minute; SAM, self‐assessment manikin (Bradley & Lang, 1994); SET, social evaluative threat; asterisks indicate the *p* values of the respective repeated measures ANOVAs, ***p < .001, **p < .01

Online measurements of adrenergic (heart rate, *F*_[1,34]_ = 38.41, *p* < .001, pupil diameter, *F*_[1,39]_ = 39.57, *p* < .001) and perceived arousal measures collected during fMRI (SAM, *F*_[1,39]_ = 23.32, *p* < .001) showed significant variations across the experiment, with significantly higher values in the SET trials compared to the control trials (Figure 2).

Similarly, repeated measures ANOVAs for the offline stress measures revealed significant variations in heart rate (*F*_[3.02,117.88]_ = 8.25, *p* < .001), systolic blood pressure (*F*_[3.38,131.69]_ = 8.57, *p* < .001), and saliva cortisol levels (*F*_[2.01,76.48]_ = 7.26, *p* = .001) across the experiment. Comparisons of assessment time points showed significant increases for all variables from T2 to T3 (i.e., before fMRI task onset and the end of Run 1; heart rate: *p* = .004, blood pressure: *p* = .001, cortisol: *p* = .001). From T3 to T4 (collected after fMRI Run 2), heart rate (*p* = .63) and systolic blood pressure (*p* = .84) remained elevated, while cortisol (*p* < .001) declined back to baseline (Figure 2).

### 3.2 Task‐associated brain activity

**Figure 3:**
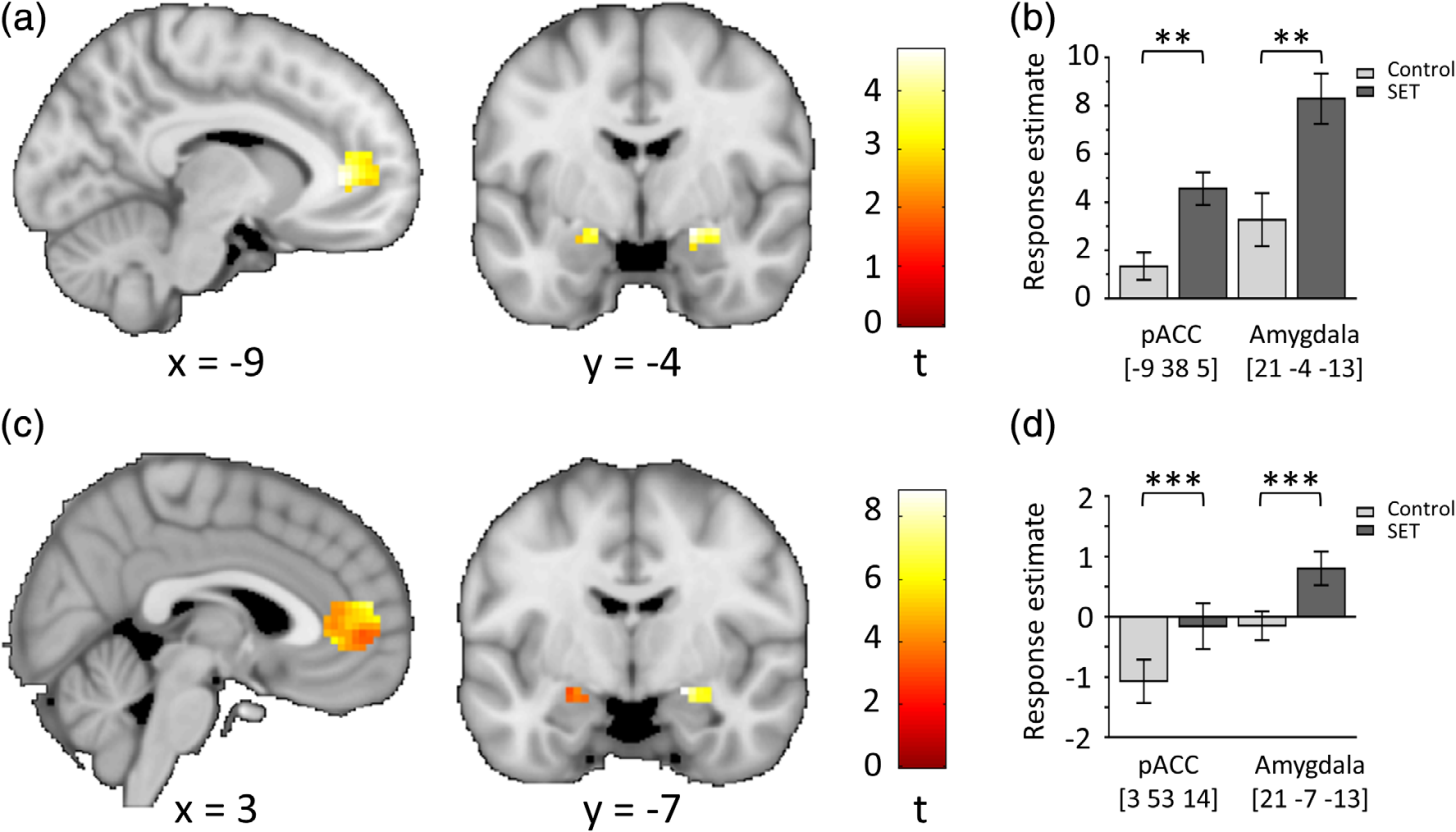
Main effects of SET on brain activation (SET > control) in the examined regions of interest. The upper panels (a, b) show data for the anticipation phase, the lower panels (c, d) for the performance phase. For presentation purposes, brain maps are displayed at *p* < .005 uncorrected for multiple comparisons. Color bars represent *t* values. b and d show estimated responses (mean ± standard error) in the peak voxels of the amygdala and pACC for the respective task phase. Coordinates are reported in standard space defined by the Montreal Neurological Institute. pACC, perigenual anterior cingulate cortex; SET, social evaluative threat

During SET anticipation and performance (contrast: SET > control), we found significantly increased activation in brain regions previously reported to be involved in SET processing (Akdeniz et al., 2014; Dedovic et al., 2005; Streit et al., 2014). Specifically, we detected significant ROI activation increases in the amygdala and pACC, for the performance phase (pACC: [3 53 14], *t* = 7.03, *p*_FWE_ < .001, amygdala: [21 −7 −13], *t* = 8.82, *p*_FWE_ < .001) and for the anticipation phase (pACC: [−9 38 5], *t* = 4.57, *p*_FWE_ = .004, amygdala: [21 −4 −13], *t* = 4.69, *p*_FWE_ = .003; Figure 3). For both amygdaIa and pACC, significant effects of condition were seen on both hemispheres (Table S1). Notably, the peak activations in the amygdala and pACC for the performance phase survived whole‐brain correction (see Figure S3 in the supplementary information for whole‐brain significant results). To evaluate the robustness of our findings, we further examined the mean activation across all voxels within the amygdala and pACC respectively, which confirmed significant activation differences between conditions for both brain regions and task phases (Figure S2). For the preparation phase, we found no significant effects within our ROI. For the reverse contrast of control > SET, there were no significant effects within our ROI in any task phase.

### 3.3 Task‐associated movement

Although we controlled for subject movement during data quality control and in our first‐level models, residual confounding of our brain activation results by potential condition‐specific differences in movement (SET vs. control) may exist, especially during the speech performance phase of the task. To exclude this possibility, we calculated and compared individual mean frame‐wise displacement estimates (Power et al., 2012) between task conditions. Notably, the analysis (Wilcoxon signed‐rank test) did not reveal any significant differences in subject movement between the SET and control conditions across the anticipation phase (*z* = −1.04, *p* = .30) and the speech performance phase (*z* = −1.23, *p* = .22) of the task. These observations argue against the confounding of our brain activation results by condition‐specific differences in movement.

### 3.4 Correlation of adrenergic arousal and brain activity

**Figure 4:**
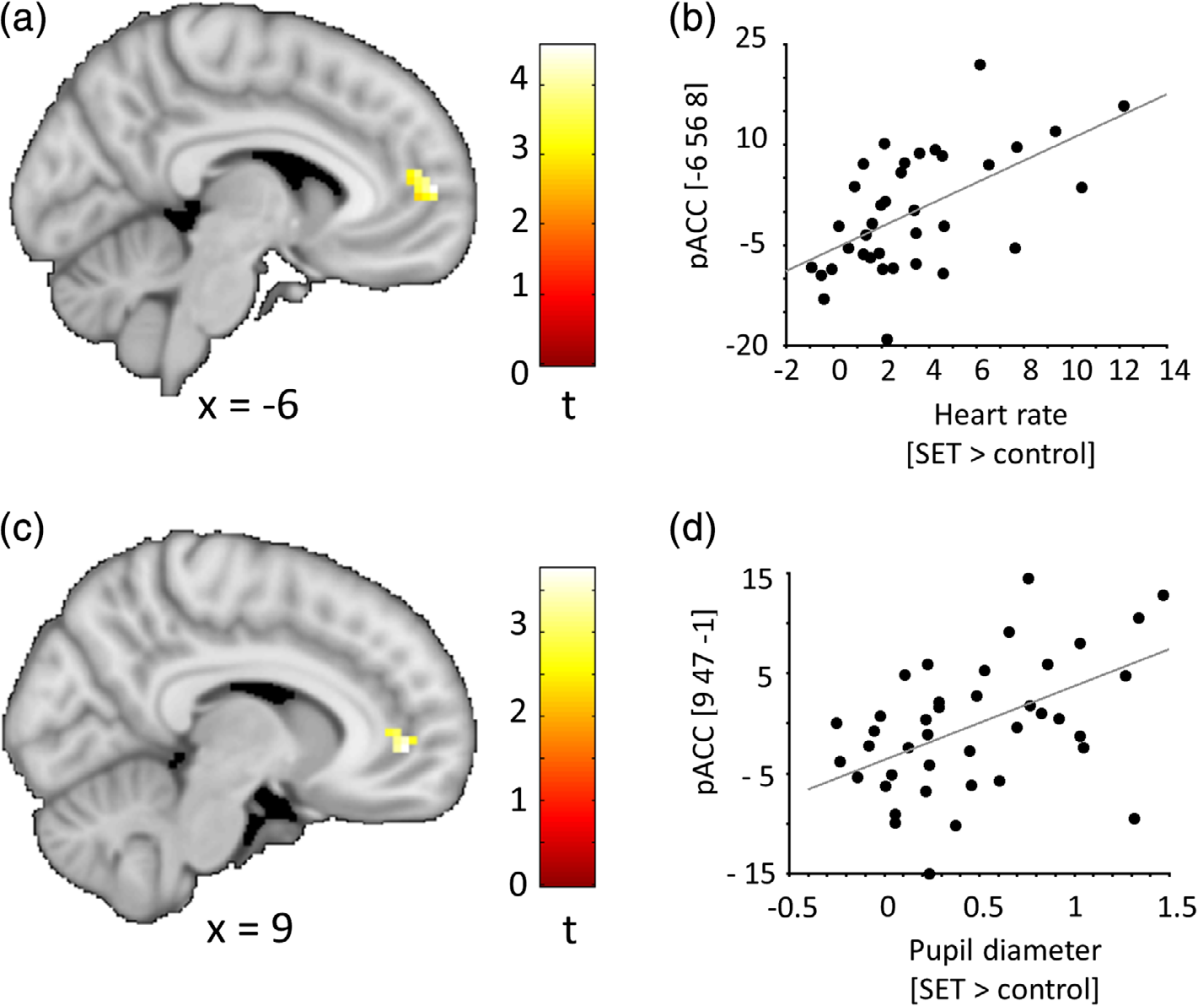
Significant associations of SET‐associated increases (SET > control) in adrenergic measures and pACC activation estimates in the anticipation phase. The upper panel (a + b) shows data for online measures of heart rate (*t* = 4.56, *p*_FWE_ = .006, ROI corrected), the lower panel (c + d) for online measures of pupil diameter (*t* = 3.75, *p*_FWE_ = .033, ROI corrected). For illustration purposes, brain maps are thresholded at *p* < .005, uncorrected. Color bars represent *t* values. Panels b and d show the regression plots for the response in the respective peak voxel. Coordinates are reported in standard space defined by the Montreal Neurological Institute. pACC, perigenual anterior cingulate cortex; SET, social evaluative threat

We found positive correlations between SET‐associated increases in online measures of adrenergic arousal and SET‐associated increases in pACC activity during the anticipation phase (heart rate: *t* = 4.56, *p*_FWE_ = .006; pupil diameter: *t* = 3.75, *p*_FWE_ = .033; both ROI‐corrected, Figure 4). No such associations were seen for the performance phase and for correlations with cortisol AUCi or SAM. Please note that tests on associations between brain responses and arousal measures were not corrected for multiple comparisons (see section 4 for details).

### 3.5 Correlation of social environmental risk measures and brain activity

**Figure 5:**
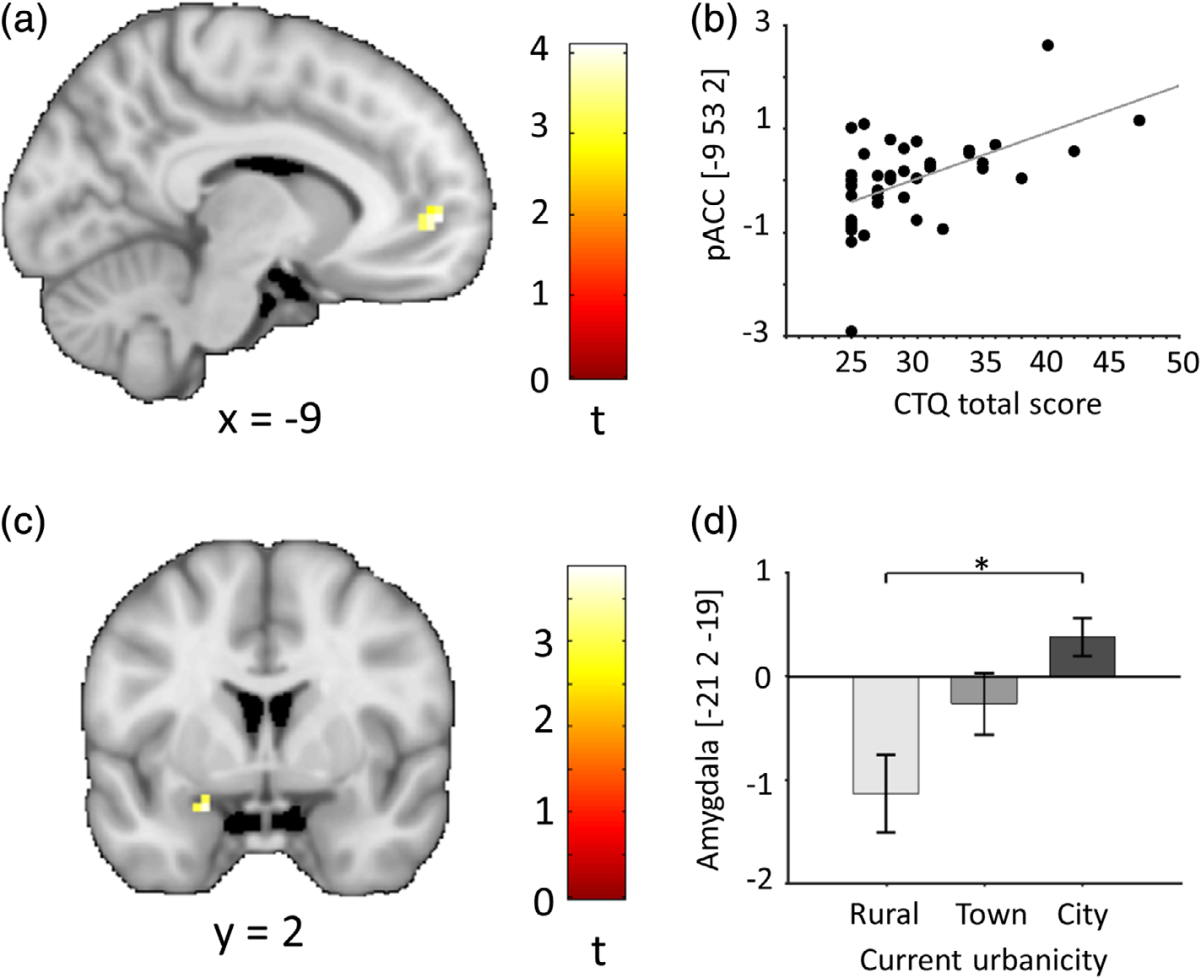
Regression of social environmental risk measures on pACC and amygdala activation estimates (SET > control) during the performance phase. Upper panel (a, b) shows data for childhood adversity (CTQ total score, *t* = 4.09, *p*_FWE_ = .016, ROI corrected), lower panel (c, d) for current urbanicity (*t* = 3.69, *p*_FWE_ = .043, ROI corrected). For illustration purposes, brain maps are thresholded at *p* < .005 uncorrected. Color bars represent *t* values. Panels b and d show plots for the response in the respective peak voxel. Statistics for childhood adversity remain significant (*t* = 4.00, *p* = .020) after post hoc exclusion of a subject with an outlier brain response in the peak voxel. Coordinates are reported in standard space defined by the Montreal Neurological Institute. pACC, perigenual anterior cingulate cortex; SET, social evaluative threat

Childhood adversity (CTQ total score, *t* = 4.09, *p*_FWE_ = .016, ROI‐corrected) and current urbanicity (*t* = 3.69, *p*_FWE_ = .043, ROI‐corrected) showed significant positive correlations with SET‐associated activity increases in pACC and amygdala, respectively, during the performance phase of the task (Figure 5). No such associations were found for urban upringing or the anticipation phase. Please note that tests on associations between brain responses and social environmental risk were not corrected for multiple comparisons (see section 4 for details).

### 3.6 Post hoc analyses of potential confounding variables

#### 3.6.1 Education

Participants of different educational levels may vary in their experiences of giving speeches and reasoned answers to questions. We, therefore, probed for associations of educational level on all variables of interest to this study and found no significant effects (all *p* > .15).

#### 3.6.2 Hormonal contraceptives

Hormonal contraceptives have been previously associated with lower cortisol responses (Kirschbaum, Kudielka, Gaab, Schommer, & Hellhammer, 1999), therefore, we probed for associations of hormonal contraceptive use in our female subgroup on all variables of interest to this study and found no significant effects (all *p* > .08).

#### 3.6.3 FMRI experience

It has been previously shown that the MR environment itself can be stressful (Muehlhan, Lueken, Wittchen, & Kirschbaum, 2011; Rampino et al., 2019). Post hoc exploratory analyses did not provide any evidence for a relevant influence of previous fMRI experience on reported results (all *p* > .17).

## 4 DISCUSSION

In this work, we present a novel fMRI paradigm to investigate the physiological, psychological, and neural correlates of the anticipation, preparation, and processing of social evaluative threat during public speaking in the absence of harsh negative investigator feedback and provoked participant performance failures. We implemented this paradigm to address some of the limitations of previous neuroimaging stress paradigms including methodological constraints of the designs, debatable ecological validity of experimental scenarios, and limited applicability of tasks in patient populations and longitudinal studies.

First, we detected a significant increase in all assessed online and offline measures of adrenergic, hormonal, and subjective arousal during the experiment. Second, we further observed significant activation increases in the pre‐hypothesized brain regions pACC and amygdala during anticipation and processing of SET, in accordance with the results of previous neuroimaging studies (Akdeniz et al., 2014; Lederbogen et al., 2011; Streit et al., 2014). Importantly, unlike in several earlier fMRI adaptations of the TSST, the experimental condition in our task was not confounded with cognitive load, we refrained from implementing any sort of harsh investigator feedback and experimentally provoked performance failures of the participants, and we thoroughly randomized the type and order of stimuli across participants and task conditions. These observations suggest that the social stress manipulation in our task, albeit relatively well‐controlled and mild compared to some previous fMRI stress tasks, was nonetheless successful in engaging the targeted regions in the neural stress regulatory circuitry while eliciting psychological and physiological responses typical for acute stress‐related arousal (Dickerson & Kemeny, 2004).

To further validate our neuroimaging results, we tested for significant relationships between SET‐associated increases in established online measures of adrenergic arousal and brain activity. Here, we detected significant positive associations of increases in heart rate and pupil diameter with pACC activity during SET anticipation. Similarly, prior studies in healthy subjects have reported associations of adrenergic arousal measures with brain activity during demanding cognitive tasks primarily in pACC (Gianaros et al., 2005, 2008, 2017; Gianaros, Jennings, et al., 2007; Wager, Waugh, et al., 2009) and have concluded that stress‐related adrenergic activity seems to be more closely related to and likely even generated by pACC rather than amygdala activity (Gianaros, Jennings, et al., 2007; Gianaros & Wager, 2015; Wager, van Ast, et al., 2009; Wager, Waugh, et al., 2009). Another reason for the more loose association of stress‐associated adrenergic measures and amygdala activation, reported in the literature, could be a faster habituation of amygdala responses in comparison to pACC (Gianaros & Wager, 2015; Plichta et al., 2014; Wager, Waugh, et al., 2009). Since our paradigm allowed for disentangling different SET task phases, and since we detected this association during SET anticipation but not during SET processing, our findings further extend this prior work by identifying SET anticipation as particularly salient for neural and adrenergic regulation of acute stress‐related arousal.

By way of external confirmation, we further examined SET‐related activation in pACC and amygdala for associations with ACE and urbanicity, two established social risk factors for psychiatric disorders. In line with our expectations, we observed a significant positive association between ACE exposure and pACC activity during SET processing in our healthy participant sample, consistent with prior studies employing emotional face processing paradigms (Fonzo et al., 2013; Hart et al., 2018; Heany et al., 2018). Moreover, as in some prior reports (Fonzo et al., 2013; Hart et al., 2018; Heany et al., 2018), we did not detect a significant relationship between ACE severity and amygdala activity. These observations are in accordance with existing neuroimaging work on ACE (Heany et al., 2018) and extend prior data by highlighting ACE associations for a social evaluative stress space processing task. Moreover, since pACC has been previously linked to other types of complex social environmental factors relevant for mental health such as ethnic minority status (Akdeniz et al., 2014) and perceived social standing (Gianaros, Horenstein et al., 2007), our data further support the assumed role of pACC as higher‐order convergence site for risk and resilience effects in the neural stress regulatory circuitry (Gianaros, Horenstein, et al., 2007; Holz et al., 2019; Meyer‐Lindenberg & Tost, 2012; Tost et al., 2015). While our data can support (but not prove) the following conclusion, it is well possible that stress induction may influence pACC and amygdala activity, for example, by altering cortisol release and corticosteroid receptor function (Boehringer et al., 2015; McEwen et al., 2015). Preclinical studies have shown that the pACC is involved in glucocorticoid‐mediated feedback inhibition of stress‐related HPA axis activity (Diorio, Viau, & Meaney, 1993). The pACC is also anatomically connected to the amygdala and assumed to regulate its activity (Paus, 2001; Pezawas et al., 2005). Amygdala activity is considered to activate the HPA axis (Herman, Ostrander, Mueller, & Figueiredo, 2005). An increased pACC activity in the absence of (a) elevated amygdala activity and (b) increased cortisol output in healthy subjects with social risk factors could reflect the necessity of increasing stress regulatory efforts to maintain a normal stress level. A more severe insufficiency of stress regulatory mechanisms, reflected in increased amygdala activity and elevated cortisol levels, which can under chronic conditions also result in amygdala atrophy and blunted cortisol responses, has been observed in patients with stress‐related psychiatric disorders (Herman et al., 2005; McEwen, 2004; Zorn et al., 2017). For urbanization, we were able to replicate increased amygdala activity during SET processing in subjects living in urban areas, a finding that has previously been observed with two other fMRI social stress paradigms (Lederbogen et al., 2011). So far, it remains unclear why some types of social risk factors directly affect amygdala function rather than pACC function also in healthy subjects. In light of the lack of any harsh investigator feedback and provoked performance failures, these observations point to the potential value of the described task for future inquiries of the neural mechanistic basis of stress‐related psychiatric disorders including in patient populations and longitudinal study designs.

Several limitations apply to our study. First, test–retest reliability studies will be necessary to confirm the applicability of our paradigm for longitudinal study designs. Second, we did not observe SET condition‐specific brain responses during speech preparation, in contrast to a study by Wager, Waugh, et al. (2009). We believe that this discrepancy may plausibly relate to differences in methods in the respective experiments: While Wager, Waugh, et al. (2009) compared a single 2‐min phase of speech preparation with 2 min of resting‐state, we compared several short speech preparation phases between task conditions that differed only in the social evaluative “load” (present or absent) of the upcoming performance phase. Furthermore, in our study, the subjects were instructed to put the same effort into the speech preparation during control trials as in experimental trials. Possibly, the subjects in our study were primarily focused on the cognitive task of speech preparation rather than on the context (SET vs. control condition) during the preparation phase. In addition, the method of preparation was not constrained in our study, that is, across subjects and trials, different strategies (e.g., autobiographic recall or visualization) may have been used, which may have reduced the probability to find significant effects across conditions during this task phase in our study. Third, our SET manipulation only resulted in a moderate adrenergic and cortisol response in healthy subjects. While this may be considered a limitation, it also suggests that we successfully reduced the intensity of the stressor by eliminating the harsh negative feedback and provoked performance failure implemented in previous fMRI adaptions of the TSST (Dickerson & Kemeny, 2004) and still yielded evidence for a noticeable adrenergic and HPA axis response in a task setting with an arguably higher ecological validity and applicability in patients. Notably, Tillfors et al. (2001, 2002) previously successfully administered a similar, but less standardized experimental approach in patients with social phobia. This encourages the applicability and implementation of our task design in clinical populations. As psychiatric disorders across diagnostic boundaries (e.g., mood and anxiety disorders and psychosis) have been linked to an altered regulation of stress responses (Zorn et al., 2017), we assume our paradigm is suitable for the investigation of a broad range of psychiatric patients. Fourth, another possible reason for the moderateness of the measured stress responses is that we included both genders in this study, and in particular, women irrespective of menstrual cycle phase. It has been shown that men exhibit higher cortisol responses to stressors than women during the follicular menstrual cycle phase or under hormonal contraceptive use (Kirschbaum et al., 1999). Notably, however, we did not observe significant differences in any of our variables of interest for hormonal contraceptive use in the female subgroup. The lack of control for menstrual cycle phase should result in lower (not higher) cortisol responses to stressors (Kirschbaum et al., 1999). Fifth, in our paradigm, we aggregated online arousal data across task phases for comparison between task conditions (SET vs. control). The reason for this was the short duration of the anticipation phase (0.5 s), which, for example, may or may not include a measurable heartbeat and thus would have been of little informative value for comparison between task phases. Sixth, correlational analyses of brain function with measures of arousal and social environmental risk were not corrected for multiple testing. The aim of the current study was to establish our novel paradigm and to examine its effect with a richness of measures. The risk of false positive findings (Type I error) within the a priori ROI was very low, considering that all brain‐level results were corrected for a bilateral ROI and that we tested for well‐known and replicated findings of stress response. We chose to explore the value of our paradigm using a number of potentially relevant measures. These are valuable by informing future work about potential hypotheses on associations to neural stress responses. However, our correlative results should be interpreted with caution until confirmed by replication. Seventh, we followed an a priori defined ROI approach to investigate psychiatric risk‐related brain areas for social stress (pACC and amygdala). Other regions as well as the temporal dynamics and coupling of areas may be of interest in future studies. Finally, a general disadvantage of stress inducing studies with sampling of control conditions on the same study day is the enduring neuroendocrine response during control trials due to the relatively slow response of the HPA axis (Ulrich‐Lai & Herman, 2009; Zschucke, Renneberg, Dimeo, Wustenberg, & Strohle, 2015).

In summary, we introduce a novel fMRI task challenging neural responses to the stressful anticipation and processing of social evaluation and validate the experiment with adrenergic, hormonal, and subjective measures of arousal. We further provide evidence for the value of the paradigm at the level of brain function by showing that key regions of the emotion and stress regulatory circuitry are engaged by the task, relate to adrenergic measures of arousal, and are influenced by social environmental risk factors for psychiatric disorders. Our study extends previous work on the neural correlates of social environmental risk factors for psychiatric disorders and provides the field with a novel experimental tool allowing for the induction of moderate and arguably ecologically more valid SET in clinical populations and in longitudinal study designs.

## CONFLICTS OF INTEREST

A.M.‐L. has received consultant fees from American Association for the Advancement of Science, Atheneum Partners, Blueprint Partnership, Boehringer Ingelheim, Daimler und Benz Stiftung, Elsevier, F. Hoffmann‐La Roche, ICARE Schizophrenia, K. G. Jebsen Foundation, L.E.K Consulting, Lundbeck International Foundation (LINF), R. Adamczak, Roche Pharma, Science Foundation, Sumitomo Dainippon Pharma, Synapsis Foundation – Alzheimer Research Switzerland, System Analytics, and has received lectures including travel fees from Boehringer Ingelheim, Fama Public Relations, Institut d'investigacions Biomèdiques August Pi i Sunyer (IDIBAPS), Janssen‐Cilag, Klinikum Christophsbad, Göppingen, Lilly Deutschland, Luzerner Psychiatrie, LVR Klinikum Düsseldorf, LWL PsychiatrieVerbund Westfalen‐Lippe, Otsuka Pharmaceuticals, Reunions i Ciencia S. L., Spanish Society of Psychiatry, Südwestrundfunk Fernsehen, Stern TV, and Vitos Klinikum Kurhessen. The remaining authors declare no conflicts of interest.

## Supporting information


**Appendix S1**. Supporting Information.

## Data Availability

Data Availability Statement: The data that support the findings of this study are available from the corresponding author upon reasonable request while inquiries are subject to EU General Data Protection Regulation.
