# Supplementary material for: Neural responses to social evaluative threat in the absence of negative investigator feedback and provoked performance failures
Source: Hum Brain Mapp. 2020 Jan 20;41(8):2092–103. doi: 10.1002/hbm.24932 (PMC7268032; doi:10.1002/hbm.24932)
Supplement: Supplementary file 1 — Appendix S1. Supporting Information. [file HBM-41-2092-s001.docx]

Fehlner P, Bilek E, Harneit A, et al. Neural responses to social evaluative threat in the absence of negative investigator feedback and provoked performance failures. Hum Brain Mapp. 2020;1–12. https://doi.org/10.1002/hbm.24932

**SUPPORTING INFORMATION**

**SUPPLEMENTAL METHODS**

**Evaluative Social Stress Paradigm**

*Speech topic item selection*

In a preparatory study for speech topic item selection, we instructed 60 volunteers fluent in German (mean [M] age and standard deviation [SD]: 32.5 ± 13.2 years; 37 females [61.7 %]) to give 81 speeches on different topics with durations between 15 s and 60 s (M = 23.89 s, SD = 8.50 s). Speech topic items were taken from English-language internet websites, aiming to prepare job applicants for selection interviews, and translated into German. For each speech topic, participants rated the expected arousal of giving a speech on the respective topic on an 11-point SAM arousal scale, ranging from 0.5 to 5.5 in steps of 0.5 (Bradley & Lang, 1994). After the speech was given the participants rated the actual arousal level. Anticipatory and actual SAM arousal ratings were significantly correlated (r = .69, p < 0.001). We selected the 56 items with the highest anticipatory arousal ratings and split them into two sets of speech topic items with identical mean and standard deviation of anticipatory SAM arousal ratings (M = 2.68, SD = 0.16). Example items are “Describe a situation in which an idea of yours was criticized.” (most arousing item: M = 5.15, SD = 1.95) and “What makes you angry?” (least arousing item: M = 3.90, SD = 1.68). Volunteers of the speech topic item selection procedure were excluded from participation in the main (fMRI) study.

List of websites used for item preselection (last retrieved 01.03.2018):

- <http://www.quintcareers.com/interview_questions.html>
- <https://www.thebalance.com/top-job-interview-questions-2061228>
- <https://www.monster.com/career-advice/article/100-potential-interview-questions>
- <http://www.indiabix.com/hr-interview/questions-and-answers/>
- <https://www.inc.com/adam-vaccaro/25-crazy-interview-questions.html>
- <https://www.glassdoor.com/blog/25-tough-interview-questions-uk-edition/>
- http://www.vault.com/blog/interviewing/19-hardest-interview-questions-on-wall-street/

*Evaluator video recording and processing*

Three distinct panels of two raters each were videotaped under identical conditions, that is, the panels had the same accessories (lab coats and blotting pad) and were videotaped with the same background scene and artificial lighting. Panel members were middle-aged to make the expert status plausible. Panel members showed serious and concentrated facial expressions, acted as if they observed and evaluated a person giving a speech, and followed regular visual prompts, for example, “raise the eyebrows” or “make notes”. Unique film material was selected for each trial. For use in the control condition, part of the footage was pixelated using a custom pixelate effect for Windows Movie Maker 6.0 (size 40; downloaded from: <http://movies.blainesville.com/2011/04/custom-pixelate-effects-for-windows.html>, last retrieved 01.03.2018). We preselected two panels for use in the fMRI study. One of these panels was randomly selected for each subject and preselected video sequences of that panel (pixelated in control trials) were shown during the performance phases of the fMRI paradigm. Post hoc analyses did not reveal any significant differences between the participants assigned to the different panels in any variable of interest to this study.

**Data Acquisition and Analysis**

*Software*

Data analyses were performed using SPM12 (www.fil.ion.ucl.ac.uk/spm/), MATLAB R2013b (http://www.mathworks.com/products/matlab/), SPSS Statistics 24 (https://www.ibm.com/), and R version 3.3.3 (https://www.r-project.org/).

*Acquisition and analysis of offline arousal measures (heart rate, blood pressure, cortisol)*

Offline arousal measures were taken at 5 time points throughout the experiment while subjects were in a supine position (see main text for details on the sampling times). Heart rate and blood pressure were collected using CAS 740 devices (CAS Medical Systems, Inc., Branford, CT, USA). Saliva samples were collected using Salivettes (Sarstedt, Nümbrecht, Germany) kept for approximately 1.5 minutes in the participant’s mouth during sampling. Saliva samples were stored at -20°C. Cortisol concentrations in saliva (in nmol/L) were quantified by the Department of Biopsychology at Technische Universität Dresden, Germany, using a luminescence-immunoassay kit (IBL, Hamburg, Germany). Samples of two subjects contained too little saliva to be analyzed. To eliminate the physiological decline in cortisol over time, we removed the linear regression of time from the data, similar to Wheelock and colleagues (Wheelock et al., 2016). Cortisol data were then logarithmized to achieve a normal distribution. As a measure of cortisol output associated with our social evaluative threat (SET) paradigm, the area under the curve with respect to increase (AUCi) was calculated for saliva samples 1 to 5 (Pruessner, Kirschbaum, Meinlschmid, & Hellhammer, 2003). At the group level, offline arousal data were analyzed using repeated-measures ANOVAs followed by post hoc tests using SPSS Statistics. When the assumption of sphericity was violated, Greenhouse-Geisser adjustments were applied. The significance threshold was set to p < 0.05.

*Acquisition and analysis of online arousal measures (heart rate, pupil diameter)*

During fMRI we continuously sampled heart rate (sampling rate: 50 Hz) using the pulse oximeter provided with the MR scanner (3T Trio; Siemens, Erlangen, Germany) and pupil diameter (sampling rate: 60 Hz) using an MR compatible camera system (camera: MRC Systems, Heidelberg, Germany; computer and software: SensoMotoric Instruments, Teltow, Germany). Data were processed (visual inspection and artifact removal) and analyzed using MATLAB. To eliminate interindividual differences in pupil size due to small differences regarding the exact position of the camera, data of each subject was z-transformed before being subjected to group analyses. For each trial the analyzed time period comprised 53 s, that is, from trial start (anticipation phase) until the end of the performance phase. At the group level, online arousal data were analyzed using repeated-measures ANOVAs to compare the experimental and control conditions using SPSS Statistics. The significance threshold was set to p < 0.05.

*Assessment of adverse childhood experiences (ACE)*

For ACE quantification we used the short version of the childhood trauma questionnaire in German language (Wingenfeld et al., 2010). This retrospective self-report measure on experiences in childhood and adolescence consists of five subscales (“emotional abuse”, “physical abuse”, “sexual abuse”, “emotional neglect”, and “physical neglect”), assessed by five items each. An example item of the physical abuse subscale is: “People in my family hit me so hard that it left me with bruises or marks.”. The 25 items are to be rated on a 5-point Likert-type scale ranging from (1) “never true” to (5) “very often true”. The minimum total score is 25 and the maximum total score 125. In our sample of healthy control subjects the mean ± SD was 29.5 ± 5.3, that is, our subjects reported a low ACE load.

*Assessment of current urbanicity and urban upbringing*

Following previous practice (Lederbogen et al., 2011; Mortensen et al., 1999), the place of residence was categorized as follows: capital (5), suburb of capital (4), cities with more than 100.000 inhabitants (3), towns with more than 10.000 inhabitants (2), and rural areas (1). Current urbanicity was defined as the category at the time of study participation. In our sample, 24 subjects lived in the city of Mannheim (category 3), 12 subjects in towns (category 2), and 5 subjects in rural areas. Urban upbringing was defined as the sum of categories multiplied by the years lived in this category from birth up to age 15. One participant raised in the capital was an outlier and therefore excluded from analyses. All other participants grew up in places of categories 1 to 3. Hence, scores of urban upbringing ranged from 15 to 45.

*MRI data acquisition and preprocessing*

A 3T Siemens Trio Scanner (Siemens, Erlangen, Germany) equipped with a 32-channel head coil was used to collect blood-oxygen-level dependent (BOLD) fMRI data (gradient-recalled echo-planar imaging, GRE-EPI, sequence: TR = 1790 ms; TE = 28 ms; 34 axially tilted slices; slice thickness = 3 mm, gap = 1 mm; in-plane resolution: 3 x 3 mm; FoV = 192 mm; flip angle = 76°; matrix: 64 x 64) and high-resolution three-dimensional anatomical images (magnetization-prepared rapid gradient-echo, MP-RAGE, sequence: TR = 2530 ms; TE = 3.8 ms; TI = 1100 ms; 176 slices; slice thickness = 1 mm; in-plane resolution: 1 x 1 mm; FoV = 256 mm; flip angle = 7°). FMRI data were processed and analyzed following standard procedures in SPM12. Preprocessing consisted of image realignment to the mean image of the time series, slice time correction, normalization to the Montreal Neurological Institute (MNI) template, resampling to 3 mm isotropic voxels and spatial smoothing with an 8 mm full-width at half-maximum Gaussian filter.

*Mask for region of interest fMRI analyses*

For region of interest (ROI) analyses, we created a single mask (1393 voxels) combining the left and right amygdala masks (468 voxels) of the Automated Anatomical Labeling Atlas (Tzourio-Mazoyer et al., 2002) and an empirically defined mask of the left and right perigenual anterior cingulate cortex (pACC; 925 voxels), depicted in Figure S1. The pACC mask was created based on the coordinates of the peak voxels (Montreal Neurological Institute, MNI, space) of previous work on the correlates of social risk and resilience for mental disorders. We calculated the center of mass [0 46 6] of the coordinates and created a sphere around it, using a diameter (12 mm) so that all of the previously reported peak coordinates were inside the mask.

List of articles accounted for in the calculation of the pACC mask:

- Akdeniz C, Schafer A, Streit F, Haller L, Wust S, Kirsch P, et al. Sex-Dependent Association of Perigenual Anterior Cingulate Cortex Volume and Migration Background, an Environmental Risk Factor for Schizophrenia. Schizophr Bull. 2017;43(4):925-34.
- Akdeniz C, Tost H, Streit F, Haddad L, Wust S, Schafer A, et al. Neuroimaging evidence for a role of neural social stress processing in ethnic minority-associated environmental risk. JAMA Psychiatry. 2014;71(6):672-80.
- Gianaros PJ, Horenstein JA, Cohen S, Matthews KA, Brown SM, Flory JD, et al. Perigenual anterior cingulate morphology covaries with perceived social standing. Soc Cogn Affect Neurosci. 2007;2(3):161-73.
- Haddad L, Schafer A, Streit F, Lederbogen F, Grimm O, Wust S, et al. Brain structure correlates of urban upbringing, an environmental risk factor for schizophrenia. Schizophr Bull. 2015;41(1):115-22.
- Lederbogen F, Kirsch P, Haddad L, Streit F, Tost H, Schuch P, et al. City living and urban upbringing affect neural social stress processing in humans. Nature. 2011;474(7352):498-501.


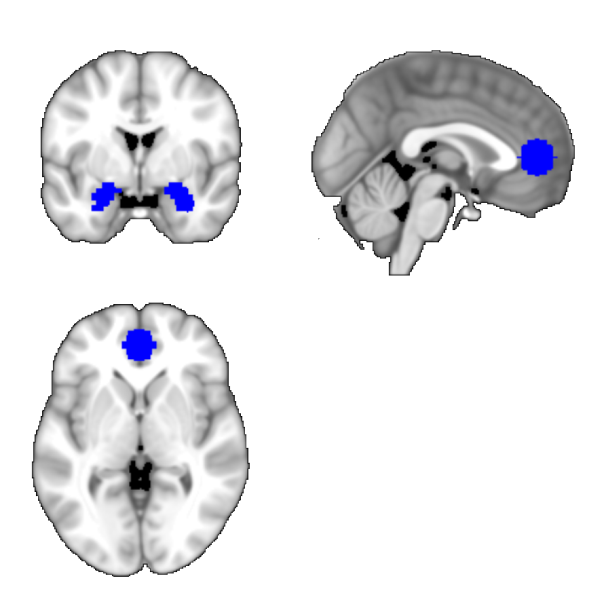


**Figure S1.** Combined amygdala and pACC mask used for region of interest (ROI) functional magnetic resonance imaging (fMRI) analyses.

**SUPPLEMENTAL RESULTS**

**Table S1.** Peak differences in brain activation (SET > control) for brain regions of interest.

| **Task phase** | **Region** |  | **Hemisphere** | **k** | **x** | **y** | **z** | **t** | **p_FWE_** |
| --- | --- | --- | --- | --- | --- | --- | --- | --- | --- |
| **Anticipation** | Amygdala |  | R | 45 | 21 | -4 | -13 | 4.69 | 0.003 |
|  | pACC |  | L | 218 | -9 | 38 | 5 | 4.57 | 0.004 |
|  | pACC |  | R |  | 6 | 50 | 14 | 4.54 | 0.004 |
|  | Amygdala |  | L | 23 | -18 | -4 | -13 | 3.80 | 0.027 |
| **Performance** | Amygdala |  | R | 63 | 21 | -7 | -13 | 8.82 | < 0.001 |
|  | pACC |  | R | 247 | 3 | 53 | 14 | 7.03 | < 0.001 |
|  | pACC |  | L |  | -9 | 38 | 8 | 5.26 | 0.001 |
|  | Amygdala |  | L | 32 | -21 | -4 | -16 | 4.04 | 0.017 |

Reported are peak voxel differences in brain activation between task conditions (SET > control) for brain regions within our ROI mask during the anticipation and performance phase, respectively. Results are family-wise error (FWE) corrected for multiple comparisons within our a priori defined ROI (bilateral amygdala and pACC, see Figure S1). Montreal Neurological Institute coordinates and statistics refer to the respective peak voxels. The cluster size k is reported for a threshold of p < 0.005 uncorrected. If k is not reported, the coordinate is a subpeak of the cluster listed above. Abbreviations: SET = social evaluative threat, ROI = region of interest, pACC = perigenual anterior cingulate cortex, L = left, R = right.


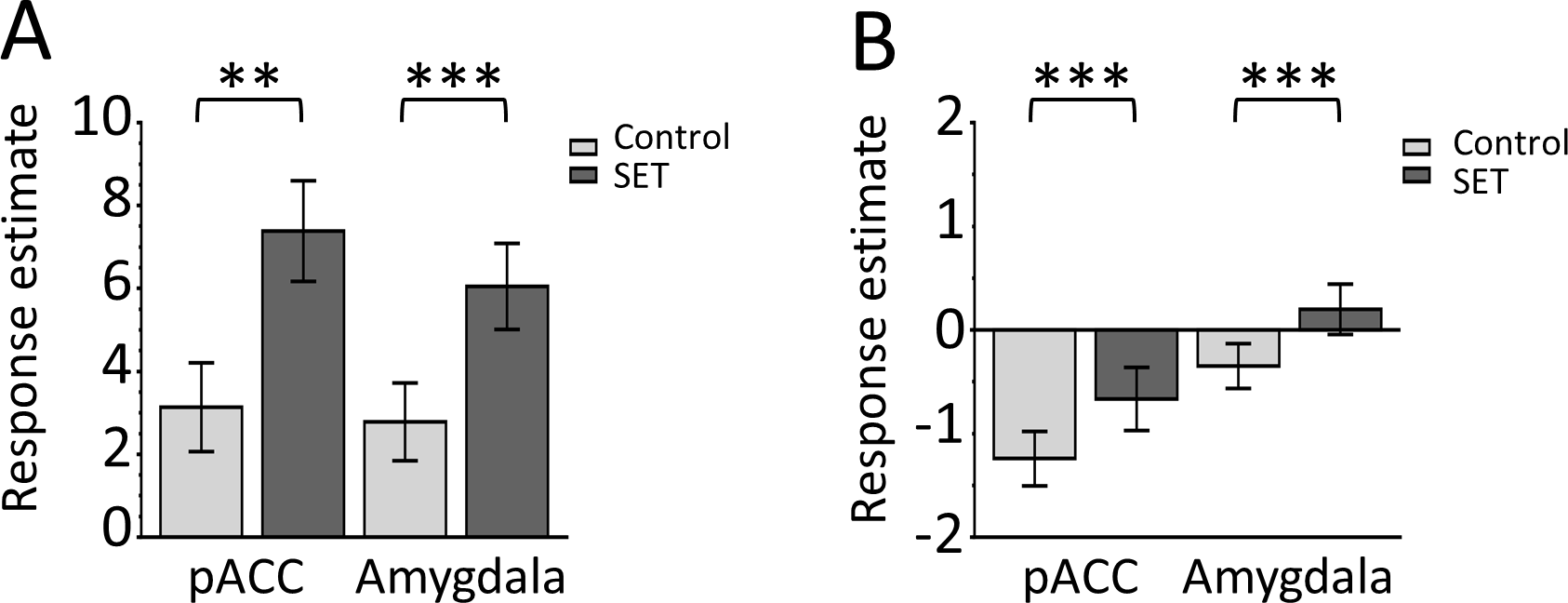


**Figure S2.** Mean response estimates (± standard error) across all voxels mapping to the bilateral amygdala and perigenual anterior cingulate cortex (pACC), respectively, for the (A) anticipation and (B) performance phase, respectively. Activation in both brain regions was significantly higher during the SET condition compared to the control condition for the anticipation (pACC: t(40) = 3.57, p = 0.001; amygdala: z = -3.95, p < 0.001) and speech performance (pACC: z = -4.46, p < 0.001; amygdala: z = -4.26, p < 0.001) phase, respectively. Asterisks indicate the p values of the respective t test or Wilcoxon signed-rank test, respectively, *** p < 0.001, ** p < 0.01. SET = social evaluative threat.


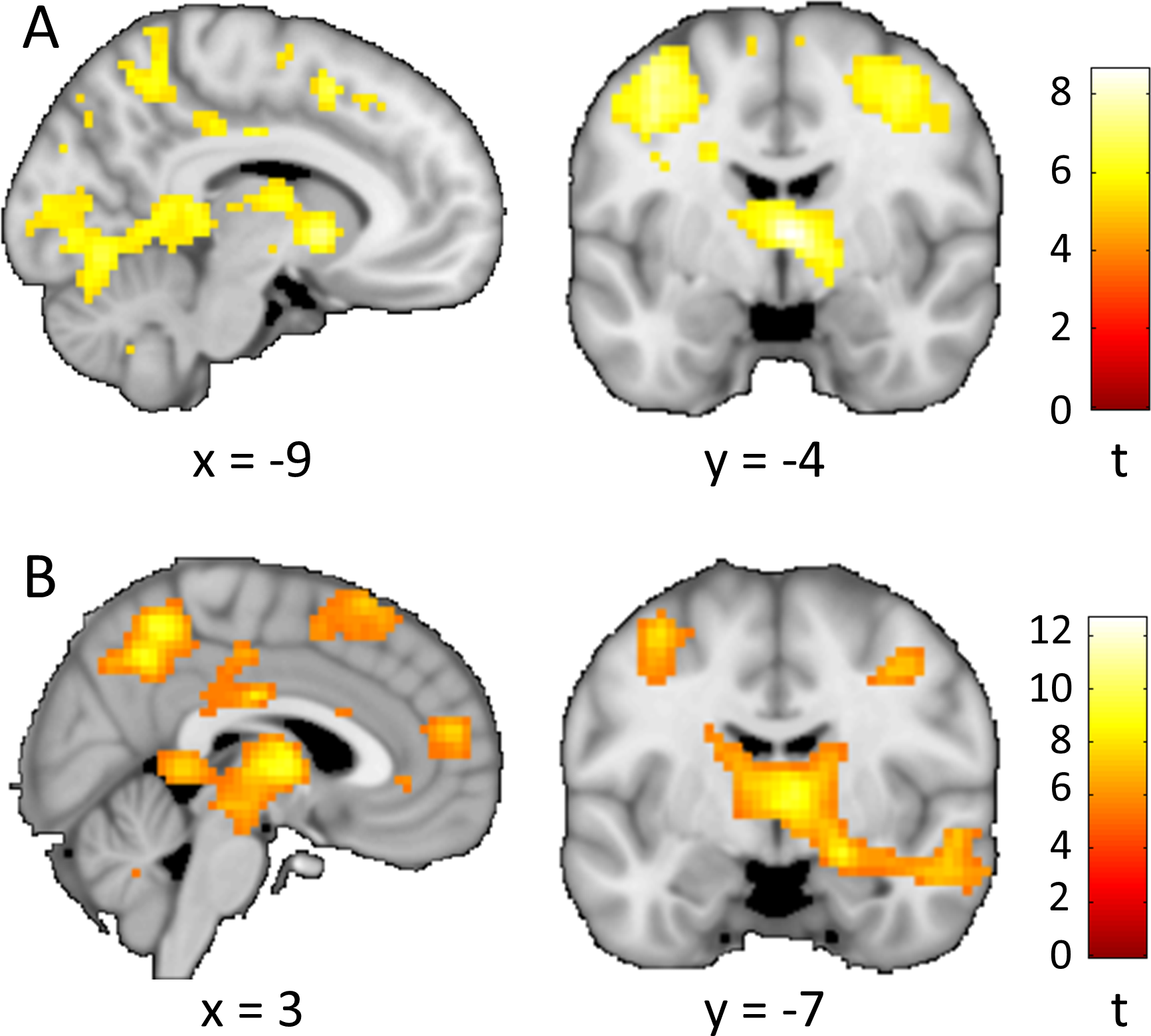


**Figure S3.** Main effects of SET on brain activation (SET > control) at p < 0.05 FWE corrected for the whole brain. A: anticipation phase (height threshold: t = 5.22), significant brain regions included the basal ganglia, thalamus, middle temporal gyrus, and insula. B: performance phase (height threshold: t = 5.28), significant brain regions included the middle and superior temporal gyri, amygdala, insula, basal ganglia, pACC, and precuneus. Color bars represent t values. Coordinates are reported in standard space defined by the Montreal Neurological Institute. Abbreviations: pACC = perigenual anterior cingulate cortex, SET = social evaluative threat.

**SUPPLEMENTAL REFERENCES**

Bradley, M. M., & Lang, P. J. (1994). Measuring emotion: the Self-Assessment Manikin and the Semantic Differential. *J Behav Ther Exp Psychiatry, 25*(1), 49-59.

Lederbogen, F., Kirsch, P., Haddad, L., Streit, F., Tost, H., Schuch, P., . . . Meyer-Lindenberg, A. (2011). City living and urban upbringing affect neural social stress processing in humans. *Nature, 474*(7352), 498-501. doi:10.1038/nature10190

Mortensen, P. B., Pedersen, C. B., Westergaard, T., Wohlfahrt, J., Ewald, H., Mors, O., . . . Melbye, M. (1999). Effects of family history and place and season of birth on the risk of schizophrenia. *N Engl J Med, 340*(8), 603-608. doi:10.1056/nejm199902253400803

Pruessner, J. C., Kirschbaum, C., Meinlschmid, G., & Hellhammer, D. H. (2003). Two formulas for computation of the area under the curve represent measures of total hormone concentration versus time-dependent change. *Psychoneuroendocrinology, 28*(7), 916-931.

Tzourio-Mazoyer, N., Landeau, B., Papathanassiou, D., Crivello, F., Etard, O., Delcroix, N., . . . Joliot, M. (2002). Automated anatomical labeling of activations in SPM using a macroscopic anatomical parcellation of the MNI MRI single-subject brain. *Neuroimage, 15*(1), 273-289. doi:10.1006/nimg.2001.0978

Wheelock, M. D., Harnett, N. G., Wood, K. H., Orem, T. R., Granger, D. A., Mrug, S., & Knight, D. C. (2016). Prefrontal Cortex Activity Is Associated with Biobehavioral Components of the Stress Response. *Front Hum Neurosci, 10*, 583. doi:10.3389/fnhum.2016.00583

Wingenfeld, K., Spitzer, C., Mensebach, C., Grabe, H. J., Hill, A., Gast, U., . . . Driessen, M. (2010). [The German Version of the Childhood Trauma Questionnaire (CTQ):Preliminary Psychometric Properties.]. *Psychother Psychosom Med Psychol, 60*(8), e13. doi:10.1055/s-0030-1253494
